# Keratinocyte-Targeted Expression of Human Laminin γ2 Rescues Skin Blistering and Early Lethality of Laminin γ2 Deficient Mice

**DOI:** 10.1371/journal.pone.0045546

**Published:** 2012-09-18

**Authors:** Tracy L. Adair-Kirk, Gail L. Griffin, Michelle J. Meyer, Diane G. Kelley, Jeffrey H. Miner, Douglas R. Keene, M. Peter Marinkovich, J. Michael Ruppert, Jouni Uitto, Robert M. Senior

**Affiliations:** 1 Division of Pulmonary and Critical Care Medicine, Department of Medicine, Washington University School of Medicine, St. Louis, Missouri, United States of America; 2 Renal Division, Department of Medicine, Washington University School of Medicine, St. Louis, Missouri, United States of America; 3 Department of Cell Biology and Physiology, Washington University School of Medicine, St. Louis, Missouri, United States of America; 4 Department of Molecular and Medical Genetics, Shriners Hospitals for Children, Portland, Oregon, United States of America; 5 Department of Dermatology, Stanford University School of Medicine, Stanford, California, United States of America; 6 Department of Biochemistry, West Virginia University School of Medicine, Morgantown, West Virginia, United States of America; 7 Dermatology and Cutaneous Biology, Jefferson Medical College, Philadelphia, Pennsylvania, United States of America; Biomedical Research Foundation of the Academy of Athens, Greece

## Abstract

Laminin-332 is a heterotrimeric basement membrane component comprised of the α3, ß3, and γ2 laminin chains. Laminin-332 modulates epithelial cell processes, such as adhesion, migration, and differentiation and is prominent in many embryonic and adult tissues. In skin, laminin-332 is secreted by keratinocytes and is a key component of hemidesmosomes connecting the keratinocytes to the underlying dermis. In mice, lack of expression of any of the three Laminin-332 chains result in impaired anchorage and detachment of the epidermis, similar to that seen in human junctional epidermolysis bullosa, and death occurs within a few days after birth. To bypass the early lethality of laminin-332 deficiency caused by the knockout of the mouse laminin γ2 chain, we expressed a dox-controllable human laminin γ2 transgene under a keratinocyte-specific promoter on the laminin γ2 (*Lamc2)* knockout background. These mice appear similar to their wild-type littermates, do not develop skin blisters, are fertile, and survive >1.5 years. Immunofluorescence analyses of the skin showed that human laminin γ2 colocalized with mouse laminin α3 and ß3 in the basement membrane zone underlying the epidermis. Furthermore, the presence of “humanized” laminin-332 in the epidermal basement membrane zone rescued the alterations in the deposition of hemidesmosomal components, such as plectin, collagen type XVII/BP180, and integrin α6 and ß4 chains, seen in conventional *Lamc2* knockout mice, leading to restored formation of hemidesmosomes. These mice will be a valuable tool for studies of organs deficient in laminin-332 and the role of laminin-332 in skin, including wound healing.

## Introduction

Skin provides a protective barrier from infection, injury, and water loss. The skin is composed of two primary layers: the epidermis, the outermost layer of skin; and the dermis, which lies just beneath the epidermis. The epidermis and dermis are separated by a thin sheet of specialized extracellular matrix called the basement membrane zone (BMZ). In addition to providing tissue boundaries and structural support, components of the basement membrane influence cell attachment, proliferation, differentiation, and migration. A defect in the structure or expression of any one of the components of the BMZ can cause tissue separation and blister formation.

Junctional epidermolysis bullosa (JEB) is one of the major forms of epidermolysis bullosa, a group of genetic skin blistering diseases. In the most severe cases, infants do not survive beyond their first year of life. JEB is most often (88%) caused by the absence of laminin (Lm)-332, due to mutations in one of the three Lm-332 chains, the α3, ß3, or γ2 chains [1]–[6]. Lm-332 is normally secreted by skin keratinocytes and is a critical component of the BMZ between the epidermis and the dermal layer [7]–[9]. Lm-332 serves as an adhesion molecule through interactions with the hemidesmosomal component integrin α6ß4 and the anchoring fibrillar component collagen VII. Most of the Lm-332 mutations that cause JEB are nonsense mutations that cause premature stop codons and result in a complete loss of Lm-332 [10]–[12].

Lm-332 has a wide tissue distribution, being deposited in epithelial basement membranes of brain, gastrointestinal tract, heart, kidney, liver, lung, trachea, skin, spleen, thymus, salivary gland, mammary gland, ovary, prostate, and testis [7], [13]–[19]. In addition to skin blistering, people with JEB experience blistering of the mucous membranes of the mouth and gastrointestinal tract, affecting nutrition. Mice with a targeted deletion of *Lama3* (laminin α3) [20] or *Lamc2* (laminin γ2) [21] genes or a spontaneous disrupting insertion of an intracisternal A particle (IAP) element in the *Lamb3* (laminin ß3) gene [22] die within a few days after birth, presumably due to the skin blistering (dehydration) or involvement of the oral and gastroesophageal mucosa (malnutrition). Unfortunately, because of the early lethality, these mice have limited experimental utility to study the role of Lm-332 in the development or repair of various tissues.

**Figure 1:**
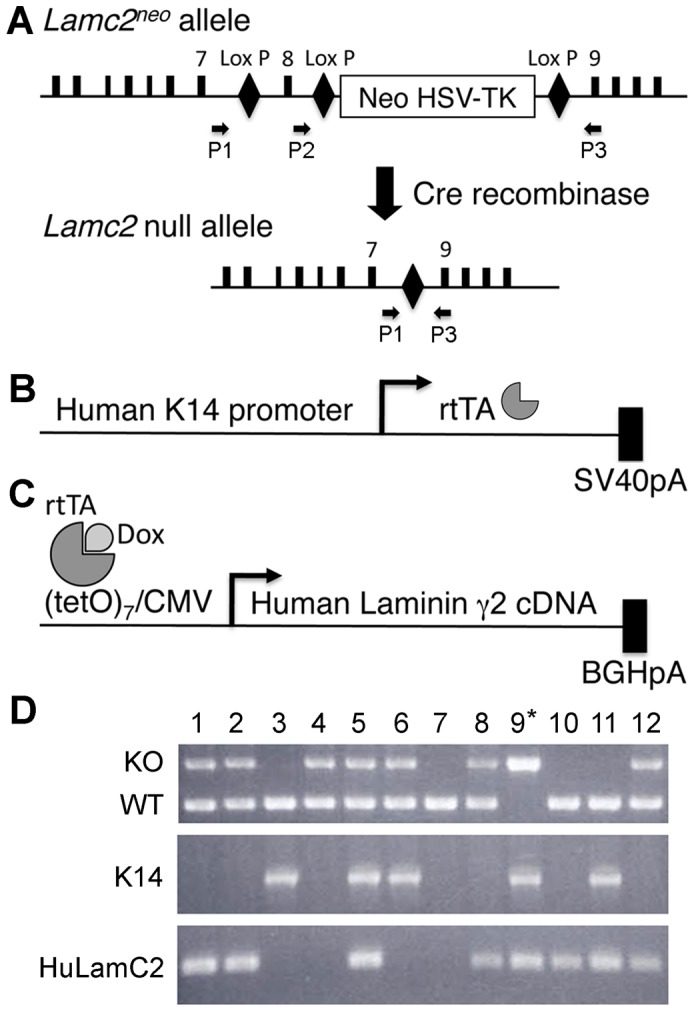
Schematic diagrams of the *Lamc2* allele and transgenes used in these studies and genotyping. (A) *Lamc2* null allele is generated by Cre recombinase, which removes exon 8 and the Neo-TK insert. Primer locations are indicated. (B) The K14-rtTA transgene contains a human keratin 14 (K14) promoter driving the reverse tetracycline transactivator (rtTA) and a SV40 poly A signal sequence. (C) The TetO-HuLamC2 transgene contains seven copies of the tetracycline operator (tetO) with a CMV minimal promoter driving the human laminin γ2 cDNA and a bovine growth hormone polyA signal sequence. The binding of doxycycline (Dox) to the rtTA promotes recruitment and binding to the tetO and activation of the promoter. (D) PCR analysis of genomic tail DNA of the *Lamc2* allele was performed using primers P1, P2, and P3. The mutant allele was detected with primer pair P1–P3, and the wild-type (WT) allele was detected using primer pair P2–P3. The K14-rtTA transgene was detected using K14- and rtTA-specific primers. The human laminin γ2 transgene was detected using primers specific to human laminin γ2. Mice that were a knockout for the *Lamc2* allele and carried both the K14-rtTA and the TetO-HuLamC2 transgenes (#9) were “rescued” *Lamc2* KO mice.

**Figure 2:**
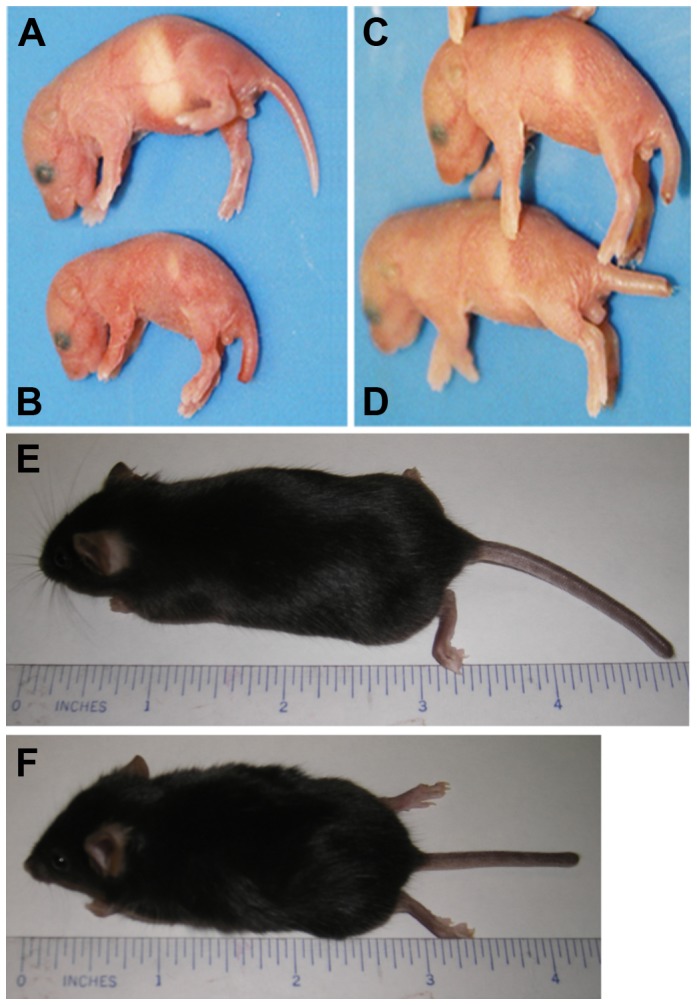
Rescued *Lamc2* KO mice appear normal at birth and live to adulthood. Images of newborn *Lamc2* Het (A), *Lamc2* KO (B), rescued *Lamc2* KO (C), and *Lamc2* WT (D), and adult *Lamc2* WT (E) and rescued *Lamc2* KO (F) are shown. Newborn *Lamc2* KO mice are occasionally smaller, have blistered feet (arrow), and a smaller milk pouch (B). *Lamc2* KO mice that carry both the K14-rtTA and TetO-HuLamC2 transgenes (C) look similar to littermate controls (A and D). Rescued *Lamc2* KO mice live to adulthood (F) and have similar length and weight as *Lamc2* WT littermates (E).

Here we generated novel tetracycline operator-regulated human laminin γ2 transgenic mice (TetO-HuLamC2), which were used in conjunction with mice carrying a keratinocyte-specific reverse tetracycline transactivator (K14-rtTA) transgene [23]–[25] to drive the expression of human laminin γ2 in keratinocytes and other keratinized stratified epithelia of *Lamc2* KO mice. Expression of the human laminin γ2 transgene specifically in the skin, tongue, and roof palate prevented the lethality of the *Lamc2* KO mice by enabling hemidesmosome formation, thus inhibiting blister formation in the skin and oral mucosa. All other tissues remained deficient in Lm-332, and yet appeared to develop grossly normal, suggesting that Lm-332 is not essential for the development of most tissues. However, this mouse could be a valuable tool to study the role of Lm-332 in repair of a variety of tissues after injury.

**Figure 3:**
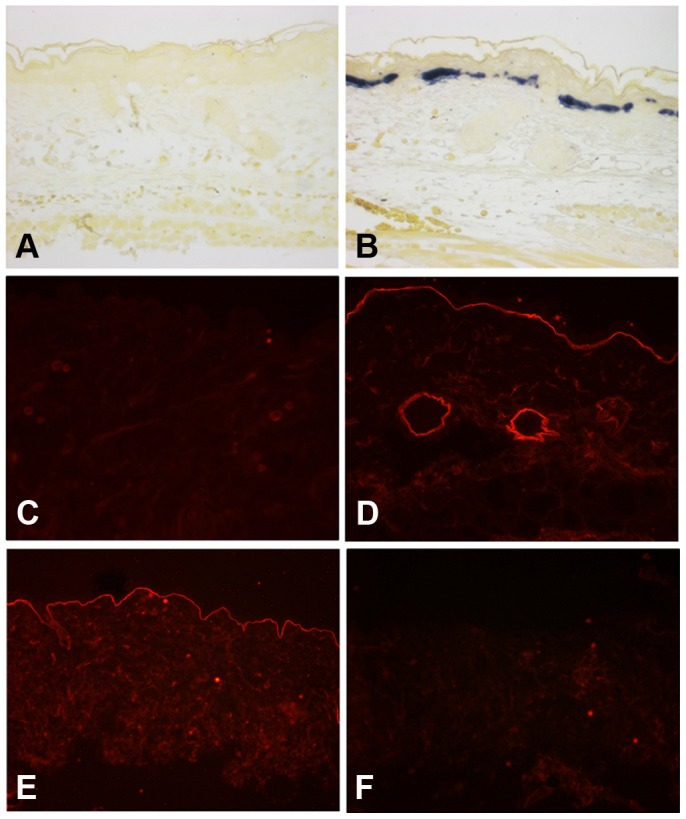
Human laminin γ2 is expressed by keratinocytes and deposited into the basement membrane. Skin tissue sections from adult *Lamc2* WT (A–C) and rescued *Lamc2* KO mice (D–F) were subjected to in situ hybridization for human laminin γ2 mRNA using a digoxigenin-labeled RNA probe (A and D) or immunostaining for human (B and E) or mouse (C and F) laminin γ2 using species-specific laminin γ2 antibodies and TRITC-conjugated secondary antibodies. Blue staining in panels A and D represents positive hybridization. Sections were counterstained with tartrazine yellow for contrast. The lack of staining in panels A and B show that the absence of human laminin γ2 expression in *Lamc2* WT mice. The lack of staining in panel F shows the absence of mouse laminin γ2 in the rescued *Lamc2* KO mice.

**Figure 4:**
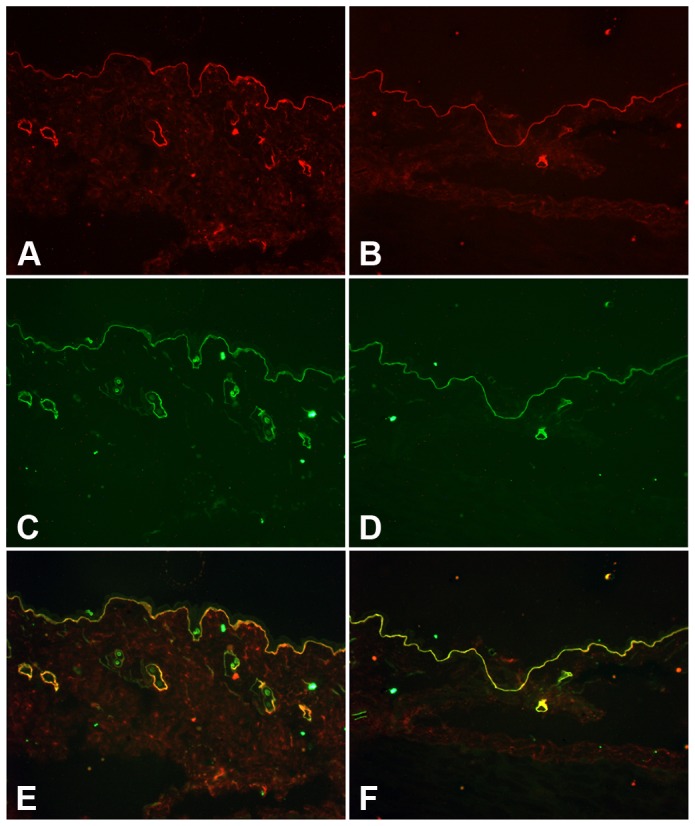
Human laminin γ2 colocalizes with mouse laminin α3 and ß3 chains in rescued *Lamc2* KO skin. Frozen skin tissue sections from adult rescued *Lamc2* KO mice were subjected to immunofluorescence staining for human laminin γ2 (A and B), mouse laminin α3 (C), and mouse laminin ß3 (D) using species-specific anti-laminin γ2 antibodies. Merged images are shown (E and F). Yellow color in panels E and F indicates colocalization.

## Materials and Methods

### Ethics Statement

All procedures using mice were approved by the Washington University School of Medicine Animal Studies Committee (protocol number: 20100104) and were performed in accordance with the Animal Welfare Act and the Guide for the Care and Use of Laboratory Animals. The mice were housed in a in a pathogen-free barrier facility within the Clinical Sciences Research Building of Washington University School of Medicine. Veterinary care was provided by the Division of Comparative Medicine at Washington University School of Medicine. Mice were provided with a surplus of food and water, and cages were changed twice a week. Mice were killed by carbon dioxide narcosis. This method was approved by the Washington University Animal Studies Committee and is consistent with the recommendations of the Panel on Euthanasia of the American Veterinary Medical Association.

**Figure 5:**
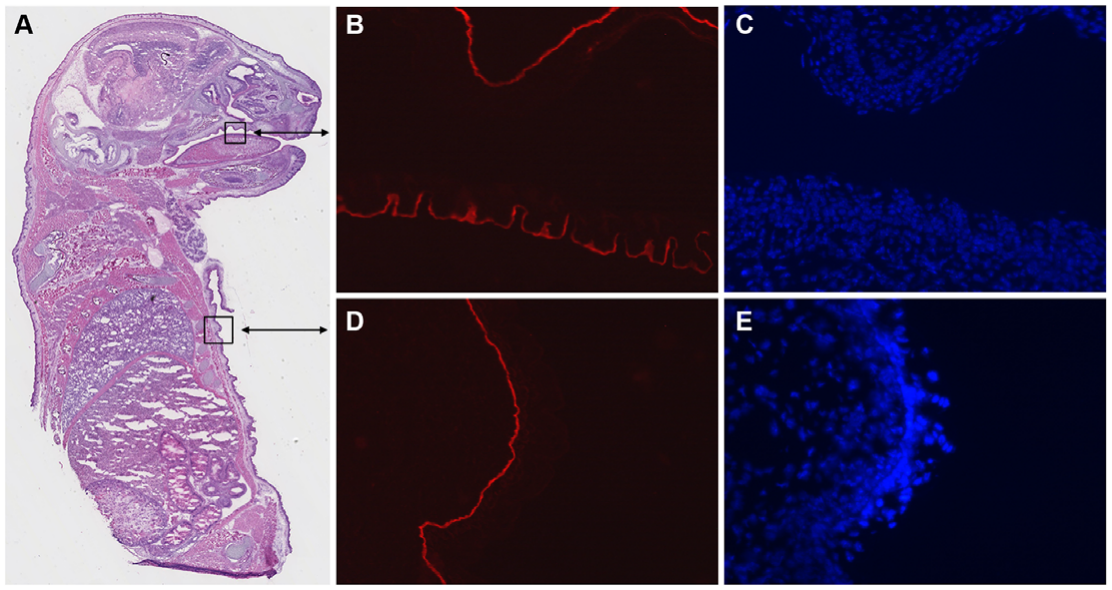
Human laminin γ2 transgene expression is restricted in the rescued *Lamc2* KO mice. Whole mount E18 rescued *Lamc2* KO embryonic tissue sections were stained with hematoxylin and eosin (H&E) (A) or for human laminin γ2 using an anti-human laminin γ2 antibody followed by TRITC-conjugated antibody (B and D). Slides were mounted with mounting media containing DAPI to allow visualization of nuclei (C and E). Human laminin γ2 was only detected in the mouth (B) and skin (D).

### Generation of “Rescued” *Lamc2* Knockout Mice

*Lamc2* knockout [21] and K14-rtTA transgenic [23]–[25] mice have been previously described. Genotyping was performed by PCR using mouse laminin γ2-specific primers (WT forward/P1 5′-CGGCTTGCTGACTTGTATCC-3′, *Lamc2* KO forward/P2 5′-AGCTAATACGGGTTCAGCC-3′, and reverse/P3 5′-TGTAACCAGAAGCACATTCC-3′) or K14-rtTA-specific primers (K14 forward 5′-GTCCGATGGGAAAGTGTAGCCTG-3′ and rtTA reverse 5′-TTTCTTCTTTAGCGACTTGATGC-3′), respectively (Figure 1A, B).

**Figure 6:**
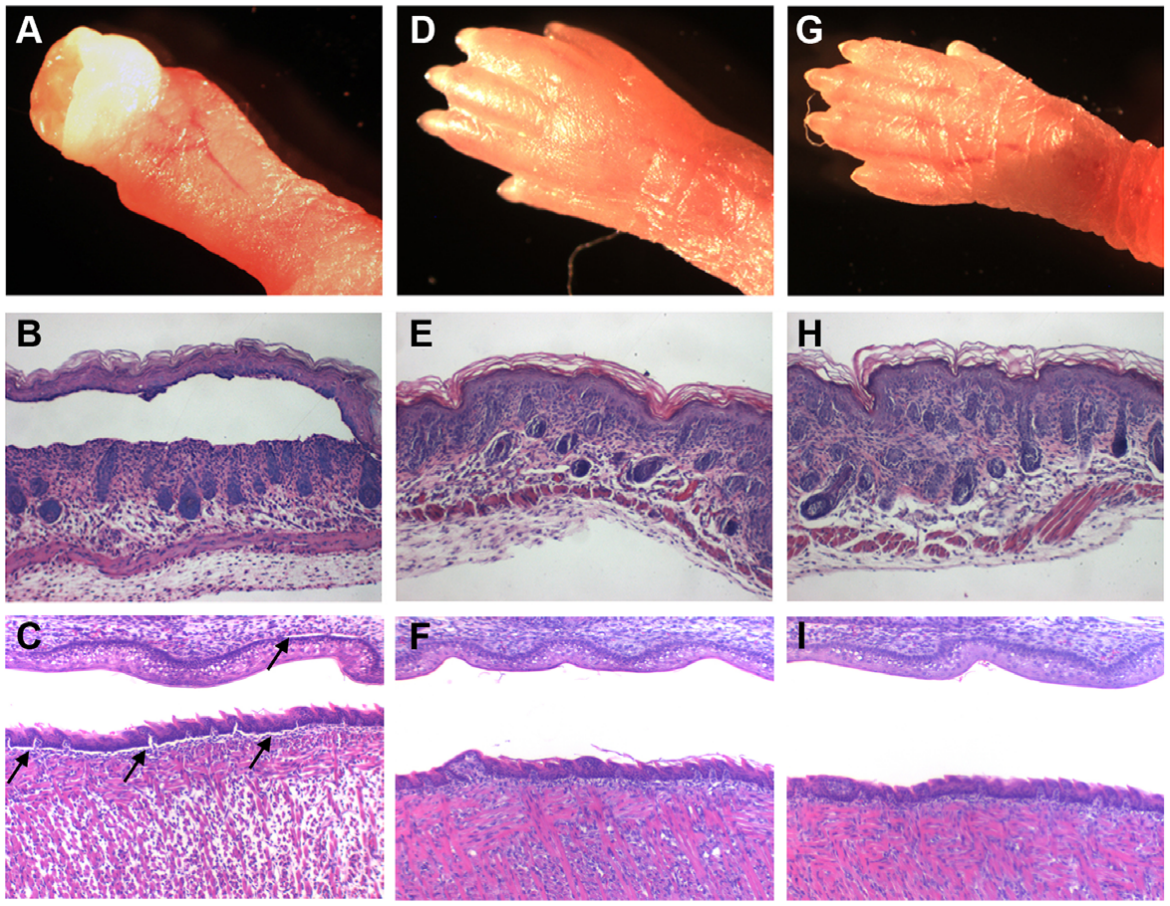
Expression of human laminin γ2 under the K14 promoter prevented blistering of rescued *Lamc2* KO mice. The paws (A, D, G), skin (B, E, H), and mouth (C, F, I) of *Lamc2* KO (A–C), rescued *Lamc2* KO (D–F), and *Lamc2* WT (G–I) newborn mice were examined. Skin blistering was most evident on the paws of *Lamc2* KO (A), but epidermal detachment (B) and separation of the oral mucosa of the roof palate and tongue (arrows in C) were detected microscopically after H&E staining. Blistering was not observed in the rescued *Lamc2* KO (D–F) or *Lamc2* WT (G–I) mice.

TetO-HuLamC2 transgenic mice were generated by microinjection of the isolated transgene (Figure 1C) into the pronuclei of C57BL/6NTac single-celled embryos. The full-length human laminin γ2 cDNA with a bovine growth hormone polyadenylation signal sequence was placed under the control of the (TetO)_7_-CMV promoter (a gift from Jeffrey Whitsett, University of Cincinnati). TetO-HuLamC2 transgenic mice were identified by PCR using human laminin γ2-specific primers (forward 5′-AGGCTGTCCAACGAAATGGG-3′ and reverse 5′-GGAGCTGTGATCCGTAGACCA-3′). Each of the 16 TetO-HuLamC2 founder lines were bred to K14-rtTA transgenic mice, and 1mg/ml doxycycline (dox) was provided in the drinking water containing 5% sucrose to induce expression of the human laminin γ2 in TetO-HuLamC2+/K14-rtTA+ double-transgenic offspring. The expression and deposition of the human laminin γ2 in the epidermal BMZ of double-transgenic offspring was examined by immunofluorescence using a human-specific anti-laminin γ2 antibody (Millipore, Billerica, MA). The offspring of two founders were maintained because they expressed the human laminin γ2 transgene in the desired fashion.

**Figure 7:**
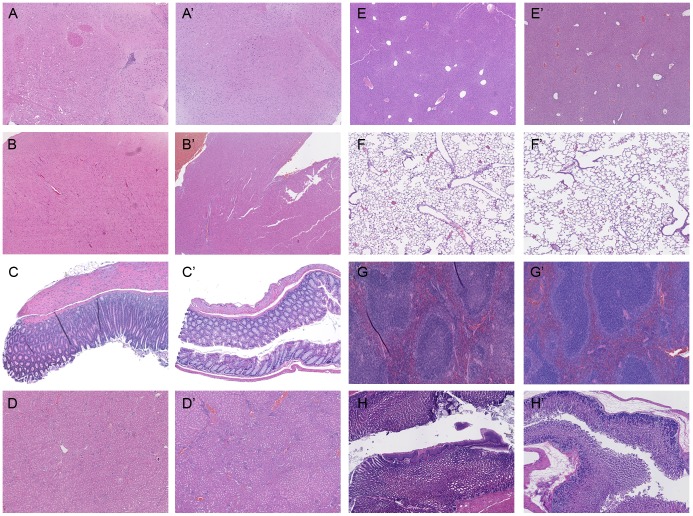
Adult tissues of rescued *Lamc2* KO mice appear grossly similar to *Lamc2* WT controls. Paraffin-embedded tissue sections of adult *Lamc2* WT (A–H) and rescued *Lamc2* KO (A'–H') mice were stained with H&E. Despite a lack of laminin γ2 expression, the brain (A, A'), heart (B, B'), intestine (C, C'), kidney (D, D'), liver (E, E'), lung (F, F'), spleen (G, G'), and stomach (H, H') appear grossly similar between the *Lamc2* WT and rescued *Lamc2* KO mice.

To generate “rescued” *Lamc2* KO mice, TetO-HuLamC2 and K14-rtTA transgenic mice were bred with *Lamc2* heterozygous mice, and the offspring were intercrossed to obtain mice that carried both TetO-HuLamC2 and K14-rtTA transgenes on a *Lamc2* KO background (TetO- *Lamc2* KO/K14-rtTA+/TetO-HuLamC2+; Figure 1D). Dox was administered at conception and continuously throughout life. Both male and female rescued *Lamc2* KO mice were fertile, which allowed interbreeding of rescued mutants to maintain the line.

**Figure 8:**
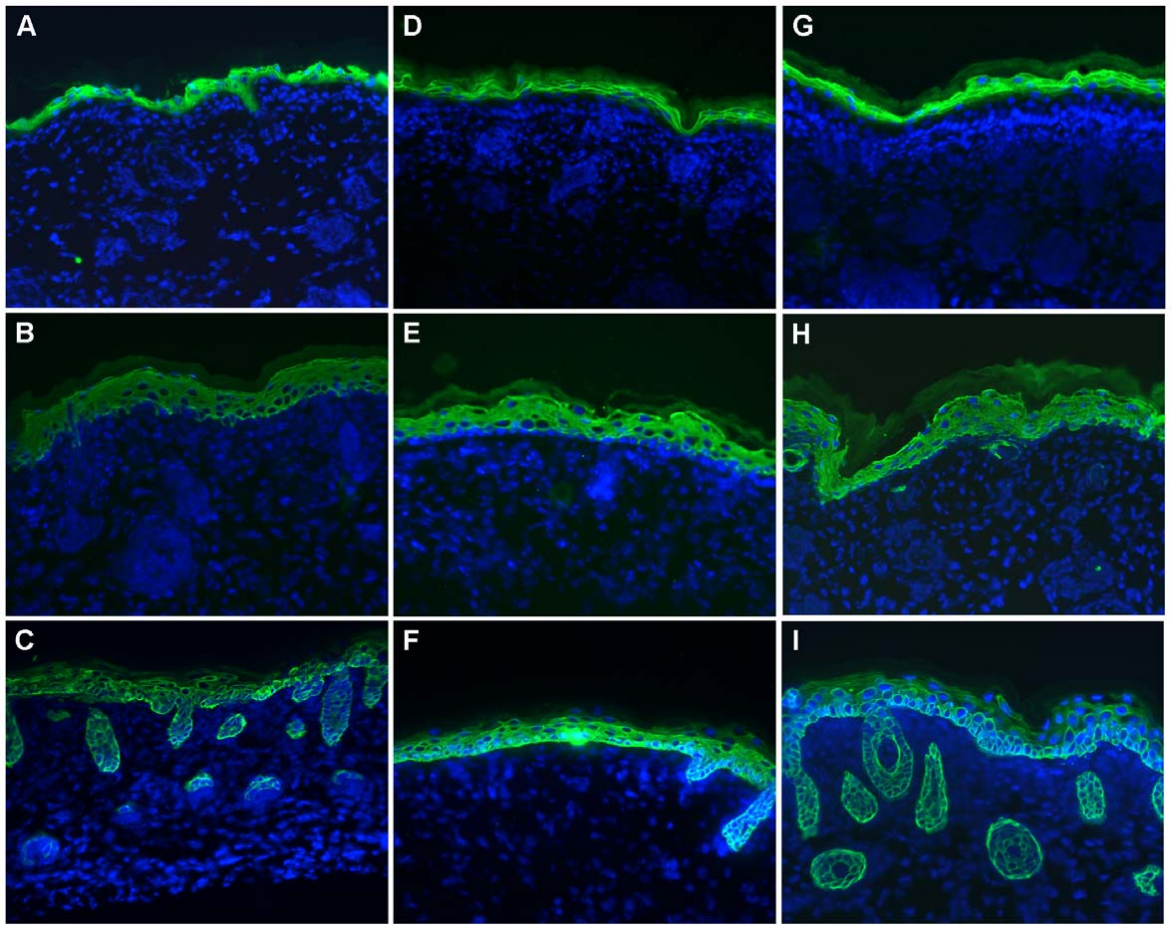
Alterations in Lm-332 expression do not alter skin differentiation. Frozen skin sections of *Lamc2* KO (A–C), rescued *Lamc2* KO (D–F), and *Lamc2* WT (G–I) newborn mice were immunostained for skin differentiation markers loricrin (A, D, G), K10 (B, E, H), and K14 (C, F, I). No significant differences were detected in the staining patterns of these skin differentiation markers in *Lamc2* KO, the rescued *Lamc2* KO, and *Lamc2* WT mice. The epidermis of each of these mice displayed loricrin in the granular layer, K10 in the spinous layer, and K14 in the basal layer.

### Histology and In Situ Hybridization

Mice were asphyxiated with CO_2_ and various tissues were fixed in 10% buffered formalin and paraffin embedded. The 5-µm sections were stained with hematoxylin and eosin (H&E) for histologic analysis by light microscopy. Images were acquired using a Nikon Optiphot II microscope and a Zeiss AxioCam HRc digital camera.

**Figure 9:**
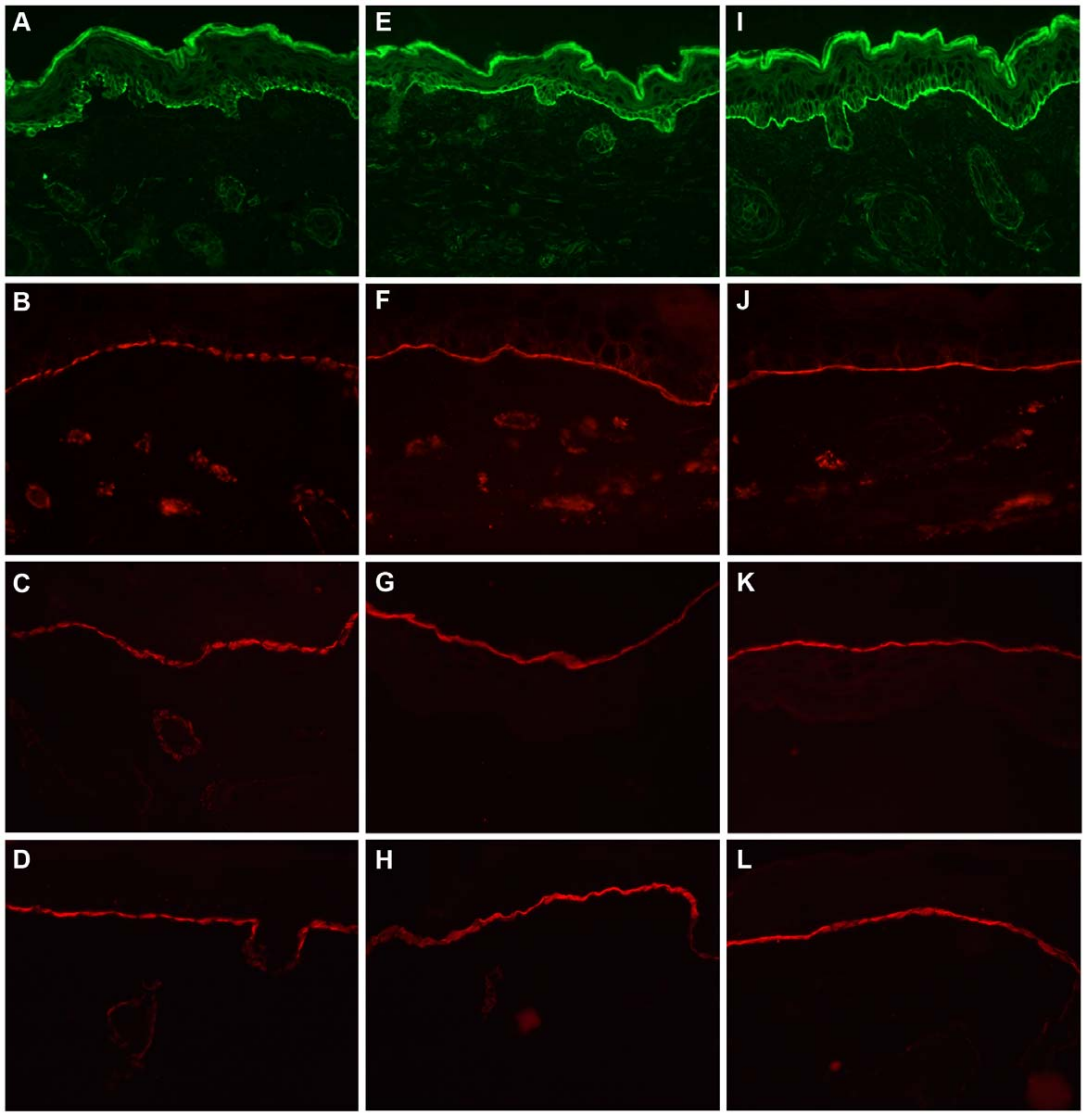
Localization of hemidesmosomal components is restored in rescued *Lamc2* KO mice. Frozen skin sections of *Lamc2* KO (A–D), rescued *Lamc2* KO (E–H), and *Lamc2* WT (I–L) newborn mice were immunostained for skin hemidesmosomal components plectin (A, E, I), BP180/Col XVII (B, F, J), and integrin chains α6 (C, G, K) and ß4 (D, H, L). The immunostaining pattern for all hemidesmosomal proteins in the *Lamc2* KO mice appeared discontinuous, whereas the staining patterns in rescued *Lamc2* KO and *Lamc2* WT mice appeared more linear.

For detection of human laminin γ2 transgene expression by in situ hybridization, a 648-bp fragment corresponding to nucleotides 2482–3129 of the human laminin γ2 gene was amplified by PCR using the full-length human laminin γ2 cDNA as the template. The resulting PCR product was subcloned into the pCRII-TOPO vector using the TOPO TA cloning kit (Invitrogen, Carlsbad, CA) as per the manufacturer's recommendations. Following vector linearization, sense and antisense digoxigenin (DIG)-labeled probe were generated using the DIG RNA Labeling Mix (Roche, Branchburg, NJ) and T7 or SP6 RNA polymerases. Hybridization of the DIG-labeled probes to 5-µm tissue sections was performed as previously described [26], and DIG was detected using the alkaline phosphate-conjugated anti-DIG antibody (Roche) and the BM purple alkaline phosphate substrate solution (Roche) as per the manufacturer's recommendations. Slides were counterstained with tartrazine yellow for contrast. The sense DIG-labeled probe was used as a negative control (data not shown).

**Figure 10:**
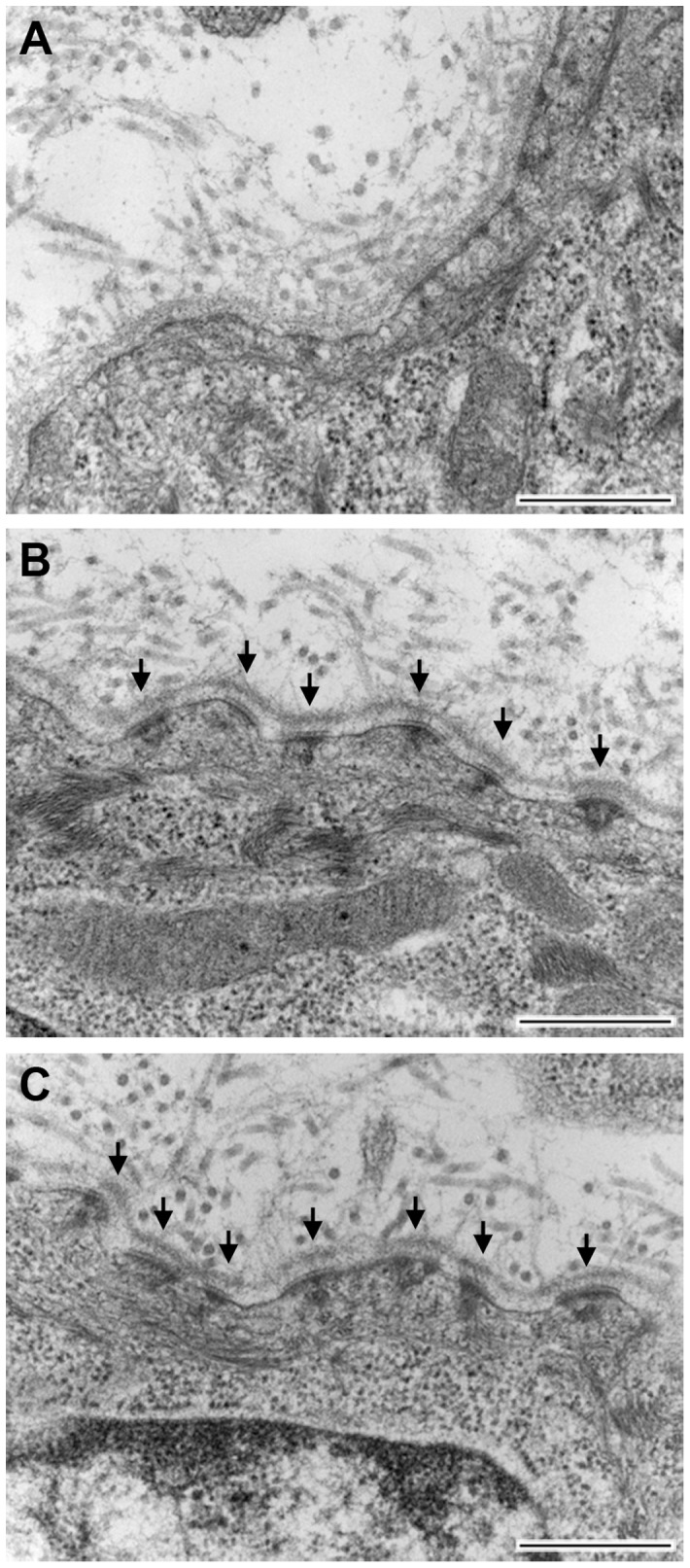
Expression of human laminin γ2 facilitates assembly of hemidesmosomes in rescued *Lamc2* KO mice. Transmission electron microscopic images of newborn skin of *Lamc2* KO (A), rescued *Lamc2* KO (B), and *Lamc2* WT (C) mice are shown. Hemidesmosomes of newborn *Lamc2* KO skin are poorly formed, devoid of lamina densa and anchoring filaments, and containing few anchoring fibrils (A). In contrast, rescued *Lamc2* KO (B) and *Lamc2* WT (C) mice had well-organized hemidesmosomes with electron dense plaques, anchoring filaments, anchoring fibrils, and darkened areas of lamina densa abutting the hemidesmosomes (arrows). All images are of the same magnification. Bar represents 500 nm.

### Immunofluorescence

Immunofluorescence analyses were performed using 5-µm frozen, non-fixed sections. Primary antibodies included: mouse laminin chains α3 and γ2 (a gift from Guerrino Meneguzzi, INSERM U634, France), and ß3 (a gift from George Plopper, Rensselaer Polytechnic Institute), human-specific laminin γ2 (Millipore), integrin chains α6 (Millipore) and ß4 (BD Biosciences), collagen XVII/BP180 (a gift from Zhi Liu, Medical College of Wisconsin), and skin markers K10, K14, loricrin (Covance, Princeton, NJ), envoplakin and plectin (Santa Cruz Biotechnology, Santa Cruz, CA). After washing, slides were incubated with FITC- or TRITC-conjugated secondary antibodies (Jackson ImmunoResearch Laboratories, West Grove, PA). Slides were mounted with Vectashield mounting media with DAPI (Vector Laboratories, Burlingame, CA).

### Electron Microscopy

Skin was prepared for electron microscopy by immersion in 1.5% glutaraldehyde/1.5% paraformaldehyde (Tousimis Research Corporation, Rockville, MD) in Dulbecco's serum-free media (SFM) containing 0.05% tannic acid for one hour followed by an extensive rinse in SFM, then post-fixation in 1% OsO_4_ for 1 hour. The samples were washed in SFM then dehydrated in a graded series of ethanol to 100%, rinsed in propylene oxide, and infiltrated in Spurrs epoxy over a total time of two hours, accelerated via microwave energy. Samples were polymerized at 70°C over 18 hours [27].

## Results

### Keratinocyte-targeted expression of human laminin γ2 prevents the early lethality of *Lamc2* KO mice

*Lamc2* KO mice exhibit blistering of the skin and oral mucosa, and die within a few days after birth [21]. To determine whether expression of laminin γ2 specifically under a keratinocyte promoter is sufficient to rescue the early lethality of *Lamc2* KO mice, we generated a new transgenic mouse line carrying the human *LAMC2* cDNA under the control of TetO-CMV regulatory element (TetO-HuLamC2, Figure 1C) to use in conjunction with mice expressing the reverse tetracycline transactivator under the control of the human keratinocyte 14 promoter (K14-rtTA, Figure 1B) [24], [25], [28] Each of these mouse lines was independently bred to *Lamc2* heterozygous mice (Figure 1A) to obtain *Lamc2* Het/TetO-HuLamC2+ and *Lamc2* Het/K14-rtTA+ mice, respectively. Then, these mice were crossed to obtain mice that carried both TetO-HuLamC2 and K14-rtTA transgenes on a *Lamc2* KO background (*Lamc2* KO/K14-rtTA+/TetO-HuLamC2+). Dox was administered at conception and continuously throughout life.

Newborn *Lamc2* KO offspring that carried neither transgene (Figure 2B) or carried only one of the transgenes (not shown) exhibited blistered skin (most notably on their paws), a smaller milk pouch, and they died within a few days after birth. These observations are consistent with previously reported findings of conventional *Lamc2* KO mice [21]. In contrast, the vast majority (>95%) of *Lamc2* KO offspring that carried both TetO-HuLamC2 and K14-rtTA transgenes (Figure 2C) appeared similar to *Lamc2* Het (Figure 2A) and *Lamc2* WT (Figure 2D) littermates at birth, and survived into adulthood (>1 year) (Figure 2F) with similar weight and length as *Lamc2* WT (Figure 2E) and *Lamc2* Het (not shown) mice. However, occasionally *Lamc2* KO/K14-rtTA+/TetO-HuLamC2+ mice were smaller than *Lamc2* Het or *Lamc2* WT mice at birth, and they remained runted as adults with no apparent affect on lifespan (data not shown). The runted phenotype was also observed in *Lamc2* KO mice (Figure 2B; [21], indicating that driving the expression of the human laminin γ2 transgene under the K14 promoter did not alter this rare phenotype.

To simplify the nomenclature for the remaining of the paper, “*Lamc2* KO” will refer to *Lamc2* KO mice that carry neither transgene or carry only one of the transgenes, and therefore do not express either the mouse or the human laminin γ2; “rescued *Lamc2* KO” will refer to *Lamc2* KO mice that carry both TetO-HuLamC2 and K14-rtTA transgenes and thus express only the human laminin γ2; and “*Lamc2* WT” will refer to mice that are wild-type at the mouse laminin γ2 allele and could carry neither transgene or carry only one of the transgenes, but not both transgenes, and thus only express the endogenous mouse laminin γ2. Both male and female rescued *Lamc2* KO mice were fertile, which allowed interbreeding to maintain the line.

### The human laminin γ2 transgene is expressed in the skin and oral mucosa of rescued *Lamc2* KO mice

The 2.3-kb fragment of the human K14 promoter has been shown to drive the expression of reporter genes and various transgenes in keratinocytes and other stratified epithelia of transgenic mice [23]–[25]. To examine the expression of the human laminin γ2 transgene, in situ hybridization and immunofluorescence analyses were performed using a human-specific laminin γ2 DIG-labeled RNA probe and a human-specific laminin γ2 antibody, respectively, on skin sections from adult rescued *Lamc2* KO mice. The human laminin γ2 transgene was not expressed by *Lamc2* WT mice that only carried the TetO-HuLamC2 transgene and not the K14-rtTA transgene (Figure 3A, 3B) or *Lamc2* WT mice that only carried the K14-rtTA transgene and not the TetO-HuLamC2 transgene (not shown). On the other hand, the human laminin γ2 transgene was expressed by basal keratinocytes of rescued *Lamc2* KO mice (Figure 3D) and deposited into the epidermal BMZ (Figure 3E). The lack of staining of mouse laminin γ2 in the rescued *Lamc2* KO mice (Figure 3F) confirmed the absence of endogenous laminin γ2 in these mice.

The deposition of the human laminin γ2 transgene in the rescued *Lamc2* KO (Figure 3E) was similar to that of mouse laminin γ2 of *Lamc2* WT mice (Figure 3C). Furthermore, the human laminin γ2 colocalized in the epidermal BMZ with endogenous mouse laminin α3 (Figure 4E) and ß3 chains (Figure 4F), suggesting that the human laminin γ2 trimerized with the mouse laminin α3 and ß3 chains to form a “humanized” Lm-332 molecule which became deposited in the epidermal BMZ.

Since mice that do not express Lm-332 die within a few days after birth, we determined when during embryonic development the human laminin γ2 transgene began to be expressed. Whole embryo tissue sections of rescued *Lamc2* KO mice from various stages of embryonic development were subjected to in situ hybridization and immunofluorescence. The human laminin γ2 transgene expression was detected by in situ hybridization in the mouth (tongue and palate) of rescued *Lamc2* KO mice as early as E14, but the laminin γ2 protein was not detected by immunofluorescence in the mouth until E16 (data not shown). The human laminin γ2 was detected in both the mouth and skin of rescued *Lamc2* KO mice at E18 (Figure 5). We did not detect human laminin γ2 in any other tissue at any stage of embryonic development or in the adult. These data confirm that the lethality of *Lamc2* KO mice can be attributed to the lack of Lm-332 expression in either the skin and/or oral mucosa.

### Expression of the Human Laminin γ2 Transgene by Rescued *Lamc2* KO Mice Prevents Blistering of the Skin and Oral Mucosa

Histopathological examination of the skin of newborn *Lamc2* KO mice that do not express human laminin γ2 showed blistering of the paws (Figures 2B and 6A) and a separation of epidermal layer from the dermis (Figure 6B). Similar separation was detected in the roof palate and tongue of *Lamc2* KO mice (Figure 6C). Driving the expression of the human laminin γ2 transgene under the control of the K14 promoter prevented epidermal detachment (Figure 6D, 6E) and mucosal epithelial separation (Figure 6F) of *Lamc2* KO mice. Images of the paws, skin, and oral mucosa of *Lamc2* WT mice are shown for comparison (Figure 6G–I). These data show that the expression of the human laminin γ2 transgene by rescued *Lamc2* KO mice prevents blistering of the skin and oral mucosa.

### Most Tissues of Adult Rescued *Lamc2* KO Mice Lack Lm-332 but Still Appear Grossly Normal

Lm-332 is a prominent laminin isoform in adult tissues [7], [13]–[19]. However, the K14 promoter drove the expression of the human laminin γ2 transgene only in the skin, tongue, and roof palate of the rescued *Lamc2* KO mice (Figure 5). Despite a lack of Lm-332 expression in most tissues of rescued *Lamc2* KO mice, histological examination of the brain, heart, intestine, kidney, liver, lung, spleen, and stomach showed that each of these tissues of rescued *Lamc2* KO mice (Figure 7A'–H') appeared grossly similar to those of *Lamc2* WT mice (Figure 7A–H). These data suggest that Lm-332 is not essential for the development of those tissues.

### Deposition of a “Humanized” Lm-332 did not Affect Epidermal Differentiation

Human laminin γ2 was detected in the epidermal BMZ of rescued *Lamc2* KO mice at E18 (Figure 5). By E18.5, the epidermis develops a fully differentiated stratified epithelium. To determine whether deposition of a “humanized” Lm-332 in the epidermal BMZ of rescued *Lamc2* KO mice alters epidermal differentiation, newborn skin sections were stained for loricrin (Figure 8A, D, G), K10 (Figure 8B, E, H), and K14 (Figure 8C, F, I). No significant differences were detected in either staining intensity or distribution of these markers in the epidermal layer of the rescued *Lamc2* KO mice (Figure 8D–F) as compared to *Lamc2* WT mice (Figure 8G–I). The epidermis of both of these mice displayed loricrin in the granular layer, K10 in the spinous layer, and K14 in the basal layer. These data suggest that substitution of mouse laminin γ2 with human laminin γ2 chain does not alter epidermal differentiation. Surprisingly, the complete absence of Lm-332 in the epidermal BMZ also did not impact epidermal differentiation. The skin of *Lamc2* KO mice (Figure 8A–C) displayed a pattern of expression and localization of loricrin, K10, and K14 similar to that observed in *Lamc2* WT mice (Figure 8G–I). These data suggest that Lm-332 is not required for epidermal differentiation.

### Expression of Human Laminin γ2 by Keratinocytes Restores Hemidesmosomes of *Lamc2* KO Mice

Hemidesmosomes are cell-extracellular matrix adhesion structures on the basal surface of keratinocytes that maintain dermal-epidermal adhesion and skin tissue integrity. Although Lm-332 is not a component of hemidesmosomes, it facilitates their assembly. To determine whether the expression of a “humanized” Lm-332 affects hemidesmosomal assembly, skin sections of newborn *Lamc2* KO, rescued *Lamc2* KO, and *Lamc2* WT mice were immunostained for hemidesmosomal components, plectin (Figure 9A, E, I), type XVII collagen/BP180 (Figure 9B, F, J), and integrin chains α6 (Figure 9C, G, K) and ß4 (Figure 9D, H, L). As seen previously [21], *Lamc2* KO mice exhibited reduced, discontinuous staining of all hemidesmosomal components on the blister roof of *Lamc2* KO mice (Figure 9A–D). In contrast, the staining patterns of plectin, collagen XVII, and integrin chains α6 and ß4 in the skin of rescued *Lamc2* KO mice (Figure 9E–H) were similar to that of *Lamc2* WT mice (Figure 9I–L). These data suggest that the expression of the human laminin γ2 transgene by rescued *Lamc2* KO mice facilitated the organization of hemidesmosomal components.

To examine the hemidesmosomes ultrastructurally, we examined the skin of newborn *Lamc2* KO, rescued *Lamc2* KO, and *Lamc2* WT mice by transmission electron microscopy. Separation of the epidermal layer of *Lamc2* KO mice was often seen. In areas where the epidermis was still attached, the hemidesmosomes were sparse and rudimentary, devoid of lamina densa and anchoring filaments, and containing few anchoring fibrils (Figure 10A). This is consistent with the findings of Meng et. al. [21]. In contrast, rescued *Lamc2* KO mice had organized hemidesmosomes with electron dense plaques, anchoring filaments, anchoring fibrils, and darkened areas of lamina densa abutting the hemidesmosomes (Figure 10B). The hemidesmosomes appeared similar in structure and density to those of *Lamc2* WT mice (Figure 10C). These data show that the expression of the human laminin γ2 transgene by rescued *Lamc2* KO mice restored hemidesmosomes which were absent in *Lamc2* KO mice.

## Discussion

Lm-332 has a wide tissue distribution and is expressed throughout development and in the adult [7], [13]–[19], suggesting that it plays an important role in the development of many tissues. People with JEB due to Lm-332 deficiency not only exhibit blistering of the skin, mouth, and digestive tract, but often display other symptoms such as hair loss, abnormalities of the fingernails, toenails, and tooth enamel, joint deformities, and difficulty breathing. This suggests that Lm-332 is also required for the development and/or maintenance of tissues other than the skin. Mice that lack the laminin γ2 chain, which is specific to the Lm-332 isoform, die within a few days after birth [21], limiting their experimental utility to study the role of Lm-332 in the development or maintenance/repair of various tissues. To bypass the lethality of the *Lamc2* KO mice, which was presumed to be due to blistering of the skin and oral mucosa, we expressed a human laminin γ2 transgene under the control of a K14 promoter previously shown to drive the expression of reporter genes/transgenes in keratinocytes and other stratified epithelia [23]–[25]. Even though this K14 promoter has driven the expression of transgenes in other tissues, such as esophagus and thymus [23]–[25], we detected human laminin γ2 only in the skin, tongue, and roof palate. As anticipated, expression of human laminin γ2 in the skin and mouth was sufficient to rescue the early postnatal lethality of *Lamc2* KO mice. The human laminin γ2 chain colocalized with the mouse α3 and ß3 chains in the basement membrane, restored hemidesmosomes, and prevented blistering of the skin and oral mucosa. These data clearly point to sites of K14 expression (i.e., skin and oral mucosa) as sites highly relevant to the early lethality of the *Lamc2* KO mice. The exact mechanism of the early postnatal death is still obscure.

Many other tissues (brain, esophagus, heart, intestine, kidney, liver, lung, spleen, stomach, and thymus) that normally express Lm-332 remained Lm-332 deficient in the rescued *Lamc2* KO mice. Despite lacking Lm-332, these tissues appeared grossly normal (Figure 7) suggesting that Lm-332 is not essential for the development of these tissues. However, it is possible that a lack of Lm-332 may have caused slight abnormalities in tissue development. For example, thorough examination of the lungs of newborn *Lamc2* KO mice revealed that the *Lamc2* KO tracheal hemidesmosomes are few and less organized and saccule size is slightly increased compared to *Lamc2* WT littermate controls [29]. Whether Lm-332 is required for later lung development could not be examined as the *Lamc2* KO mice die before alveolarization occurs. Furthermore, recently a hypomorphic laminin γ2 mouse, due to a spontaneous insertion of murine leukemia virus long terminal repeat, has been discovered that progressively develops JEB-like signs of disease including skin blisters, loss of bone mineralization, abnormal teeth, and decreased lung function (lower pressure-volume curves) [30]. A more in-depth examination of each tissue of the rescued *Lamc2* KO mice is needed. In addition, since Lm-332 modulates cellular functions involved in wound healing, such as cell attachment, migration, proliferation, and differentiation, it is possible that repair following injury of these tissues may be affected without Lm-332.

JEB is an inherited skin blistering disorder most often caused by nonsense mutations in the laminin α3, ß3, or γ2 chains, resulting in a complete loss of Lm-332 expression [10]–[12]. Recurrent or persistent erosions of the epidermal surface render afflicted individuals susceptible to serious infections, often resulting in premature death. Wound healing occurs in patients with JEB, albeit delayed and often with persistent granulation tissue. Studying animal models of JEB due to Lm-332 deficiency may provide insights into the pathogenic mechanisms by which JEB wounds heal. However, mice that completely lack Lm-332 die within a few days after birth [20]–[22], limiting their experimental utility. Other mouse models of JEB include the Col17a1 KO that had 20% survival into adulthood [31], [32] and a spontaneous laminin γ2 hypomorphic mouse [30]. However, the ideal animal model to study JEB blister/wound repair would recapitulate the BMZ with an absence of Lm-332, which occurs in the majority of JEB patients. The mice developed in this study can provide a model of wound repair without Lm-332. By driving the expression of a human laminin γ2 transgene in a keratinocyte-specific, dox-controllable manner, we can prevent the skin blistering and early postnatal lethality of *Lamc2* KO mice. However, after dox withdrawal and subsequent loss of Lm-332 expression in the adult rescued *Lamc2* KO mice, these mice can be useful for studies of Lm-332 in skin, including wound healing and possibly blister formation.

In summary, we have generated dox-controllable human laminin γ2 transgenic mice, which were used in these studies to rescue *Lamc2* KO mice by driving expression via the K14 promoter in the skin and oral mucosa. The “humanized” Lm-332 was deposited in the basement membrane, restored hemidesmosomes, prevented blistering of the skin and oral mucosa, and promoted survival of *Lamc2* KO mice into adulthood. Because the expression was limited to the skin and mouth, the rescued *Lamc2* KO mice will be valuable for studies of Lm-332 deficiency in many organs. In addition, the dox-controllable element of this system will facilitate studies of Lm-332 in skin, including wound healing.
